# The first report of autochthonous non-vector-borne transmission of canine leishmaniosis in the Nordic countries

**DOI:** 10.1186/s13028-014-0084-9

**Published:** 2014-12-10

**Authors:** Veera Karkamo, Anu Kaistinen, Anu Näreaho, Kati Dillard, Katri Vainio-Siukola, Gabriele Vidgrén, Niina Tuoresmäki, Marjukka Anttila

**Affiliations:** Finnish Food Safety Authority Evira, Pathology Research Unit, Mustialankatu 3, FI-00790 Helsinki, Finland; Veterinary Clinic Tähti, Kypärätie 19, FI-40630 Jyväskylä, Finland; Faculty of Veterinary Medicine, Department of Basic Veterinary Sciences, University of Helsinki, Agnes Sjöberginkatu 2, FI-00790 Helsinki, Finland; Movet Oy, Bioteknia 1, Neulaniementie 2, FI-70210 Kuopio, Finland

**Keywords:** Canine leishmaniosis, Venereal/horizontal/vertical transmission, Glomerulonephritis, Boxer dog, Zoonosis, Dog bites

## Abstract

**Background:**

*Leishmania* spp. are zoonotic protozoans that infect humans and other mammals such as dogs. The most significant causative species in dogs is *L. infantum*. In dogs, leishmaniosis is a potentially progressive, chronic disease with varying clinical outcomes. Autochthonous cases of canine leishmaniosis have not previously been reported in the Nordic countries.

**Results:**

In this report we describe the first diagnosed autochthonous cases of canine leishmaniosis in Finland, in which transmission via a suitable arthropod vector was absent. Two Finnish boxers that had never been in endemic areas of *Leishmania* spp., had never received blood transfusions, nor were infested by ectoparasites were diagnosed with leishmaniosis. Another dog was found with elevated *Leishmania* antibodies. A fourth boxer dog that had been in Spain was considered to be the source of these infections. Transmission occurred through biting wounds and semen, however, transplacental infection in one of the dogs could not be ruled out.

Two of the infected dogs developed a serious disease and were euthanized and sent for necropsy. The first one suffered from membranoproliferative glomerulonephritis and the second one had a chronic systemic disease. *Leishmania* sp. was detected from tissues by PCR and/or IHC in both dogs. The third infected dog was serologically positive for *Leishmania* sp. but remained free of clinical signs.

**Conclusions:**

This case report shows that imported *Leishmania*-infected dogs may pose a risk for domestic dogs, even without suitable local arthropod vectors.

## Background

*Leishmania* spp. are protozoan parasites that infect humans, dogs and other mammals [1,2]. There are over 20 different species of *Leishmania*, and at least 12 species have been reported to infect dogs [3]. Endemic areas of leishmaniosis are the Mediterranean area in Europe, the Middle East, Far East and parts of Africa, and Middle and South America [2-4].

Sandflies, *Phlebotomus* spp. in Europe and *Lutzomyia* spp. in South America, are the natural vectors of *Leishmania* spp. [5,6]. The parasites multiply in the sandflies’ midgut by binary fission. The sandflies transmit the infection to the vertebrate host when sucking blood, and the protozoa assume an intracellular amastigote form [7]. Some other hematophagous arthropods such as ticks and fleas may act as vectors for *Leishmania* spp. [8,9].

*Leishmania* spp. are zoonotic agents that may cause a serious and potentially life-threatening disease in humans [4]. Asymptomatic human carriers are reported in controversial ratios [3]. Dogs are considered to be the main reservoir hosts of *Leishmania* spp. in the domestic animal environment [10].

Canine leishmaniosis is a progressive, chronic disease with varying clinical outcomes. The most important species is *L. infantum* (syn. *L. chagasi*) [1,11]. The clinical course varies from an asymptomatic infection to a cutaneous form and a life-threatening generalized visceral disease with various clinical signs, usually affecting the skin, lymphatic organs and hematopoietic system. Resistant individuals are able to mount an effective cell-mediated response. The clinical disease is usually treatable if diagnosed early, but often results in a carrier state [1,2,11,12]. Susceptibility varies among dog breeds, and boxer dogs are found to be particularly susceptible to the infection [13].

In addition to being transmitted via sandflies and other hematophagous vectors, *Leishmania* spp. can spread vertically [14,15], venereally [16,17] and via blood transfusions [18,19]. *Leishmania* spp. are shed intermittently to semen and transmitted from infected males to females with a variable rate during mating [16]. Further, transmission through dog bites has been suspected [20].

Due to the guarded prognosis of leishmaniosis both in people and in animals, it is important to control the vector populations and prevent the infection. Control measures used include the use of repellents and other measures to decrease the level of vector contacts, euthanasia of infected animals and development of more effective vaccines and drugs [21].

The frequency of infection depends on several factors including the nature of the habitat, the population densities of dogs and vectors, the presence of other possible hosts, the level of exposure to vectors and the proper use of vector repellents [22,23].

Leishmaniosis is spreading geographically towards the north, especially in the Western hemisphere, but also in Europe. This is probably related to the climate change, which may influence the habitate of its vectors, especially sandflies. Leishmaniosis adopts a seasonal pattern according to the local climate and in Europe it is spreading at least via *Phlebotomus* spp. [20,24-29]. In addition, movement of pets across borders is becoming increasingly common and may result in the spread of many infectious diseases, such as leishmaniosis [30].

In this report we describe the first autochthonous cases of dog-to-dog transmission of canine leishmaniosis in the Nordic countries.

## Case presentation

### Study population and history

**Figure 1:**
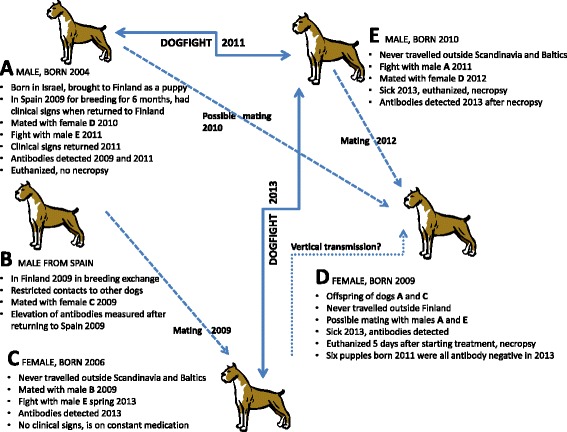
**Schematic presentation of the events related to the case study.** (Dogs **A** to **E**).

Five boxer dogs (A-E) were involved in this investigation (Figure 1). Males A & E and females C & D lived in one kennel in Finland, while male B lived in Spain. Male A stayed in Spain for six months in 2009 for breeding purposes, while male B stayed in the Finnish kennel as exchange. Male B was mated with female C but otherwise kept apart from the other dogs in the kennel. Female C gave birth in 2009 to a litter that included female D. Ectoparasites that could have been involved in the spread of infections were not observed in the kennel during the time of the investigation.

#### Male A

Male A had been examined by a veterinarian before travelling to Spain and was found clinically healthy. However, upon his return to Finland in November 2009 he had lost weight, suffered from polydipsia, polyuria and balanitis, and had a dull coat, scaly skin and moderate muscle atrophy. Polymerase chain reaction (PCR) for *Leishmania* sp. in blood was negative, while serology for *Leishmania* spp. antibodies was positive (12.9%; reference values: <7% negative, 7–12% borderline, >12% positive) (VetMedLabor, Leipzig, Germany). Although no anti-*Leishmania* treatment was given, the skin lesions resolved following treatment with topical antimicrobial ointment and a medical shampoo. However, the condition of male A began to worsen in August 2011 with loss of appetite and polydipsia and at a clinical examination in September 2011, skin lesions had returned and had become more severe. Serology revealed a highly elevated antibody level (74.5%) and a subsequent blood PCR showed presence of *Leishmania* spp. The condition worsened despite anti-*Leishmania* treatment and the dog was subsequently euthanized.

During the period since the return from Spain, he possibly accidentally mated with female D (June 2010) but (the unconfirmed) pregnancy was medically interrupted.

During the disease progression (August 2011), male A had a fight with male E, which resulted in wounds in both males. Later (spring 2013) male E and female C had a fight that injured both dogs.

#### Male E

Male E developed signs of impaired physical state with moderate general muscle atrophy and edema in the front legs and hind legs (below the hocks) in August 2013. Uremia and highly elevated blood creatinine value were diagnosed and the urine contained moderate amounts of blood, protein and *Escherichia coli*. The condition worsened despite intensive treatment with antibiotics and male E was subsequently euthanized.

Male E accidentally mated with female D in June 2012, i.e. almost 1 year after he had got biting wounds from male A but before developing disease. Female D became pregnant but the pregnancy was medically interrupted.

Male E’s exposure to *Leishmania* sp. was retrospectively re-evaluated by ELISA (*Leishmania*-ELISA Dog, Afosa GmbH Dahlewitz, Germany) based on a stored serum sample taken earlier. The antibody level was found highly elevated, i.e. 43.3% (reference values: <7% negative, 7–12% borderline, >12% positive).

#### Female D

Female D was diagnosed with dermatitis in September 2013. The skin lesions were characterized by periocular alopecia, inflamed grooves around the nose and a few scabs on the top of the head. Leishmaniosis was not considered as she had never travelled outside of Finland. Her condition worsened within the next months with weight loss, polydipsia, polyuria, systolic murmur in the mitral valve area, moderately enlarged pharyngeal and subscapular lymph nodes, and progression of the dermatitis. Serology for anti-*L. infantum* antibodies was positive, 65% (Afosa GmbH Dahlewitz). Anti-*Leishmania* treatment was initiated but female D was was euthanized after 5 days due to worsening condition.

Further, at this time, female C was tested and she had a slightly elevated antibody level (7.9%). She was put on an allopurinol treatment and has remained free of clinical signs.

#### Male B

The Spanish male B has remained clinically normal during the entire period since his stay in Finland. However, he tested positive for *Leishmania* spp. antibodies after returning to Spain (more specific data not available).

### Pathology

Male E and female D were submitted for necropsy. Complete necropsy was performed and samples for histological evaluation were taken from all of the major organs, the bone marrow and gross lesions. Tissues were fixed in neutral-buffered 10% formalin, routinely processed, embedded in paraffin, sectioned at 4 μm, and stained with hematoxylin and eosin (HE). Kidney sections were also stained with Congo red for amyloid, Masson’s trichrome for collagen and fibrosis, periodic acid-Schiff stain for connective tissue carbohydrates, and Jones’ methenamine silver stain for basement membrane. Samples of female D’s skin, bone marrow, lymph nodes and the spleen were additionally stained with Giemsa for parasites.

The only macroscopic findings in male E were anemia and pale, swollen kidneys. Histologically, the findings were consistent with membranoproliferative glomerulonephritis and proteinuria. There were no histopathological changes in the other organs, including the bone marrow.

The most prominent lesions in female D were present in the skin of the ear pinnae. Bilaterally, on the outside of the pinnae, there was a crusty lesion along the margins of the ear lobes extending towards the center and the hair coat was diffusely thinned. The bone marrow was deep red, the parotid lymph nodes were moderately enlarged and all of the lymph nodes examined were slightly larger than normal.

**Figure 2:**
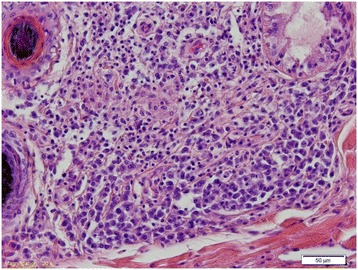
**Histological section of the skin of the ear pinna from female D.** There is marked perifollicular to interstitial inflammatory infiltrate consisting of plasma cells and macrophages. There is brownish granular intracytoplasmic material in the macrophages but no obvious organisms are present. Hematoxylin and eosin (HE) stain.

**Figure 3:**
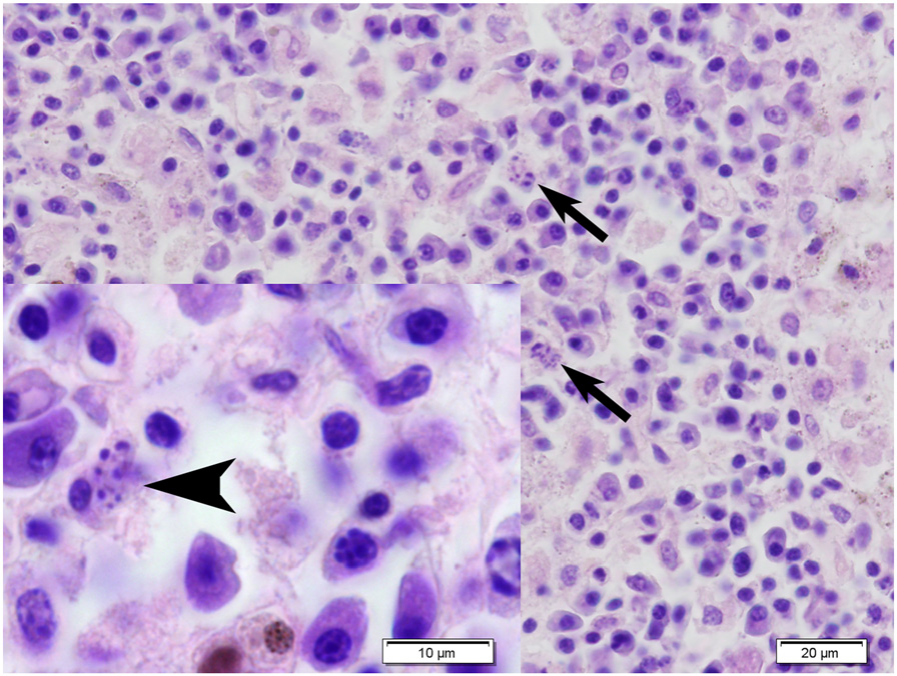
**Histological section of a lymph node from female D.** The medulla contained numerous plasma cells and macrophages. Within the cytoplasm of some of the macrophages there were variably sized oval to round particles (arrows and arrow head). Kinetoplasts typical for *Leishmania* sp. were not seen. Hematoxylin and eosin (HE) stain.

Histologically, there was a marked interstitial and perifollicular infiltrate consisting of lymphocytes and plasma cells with a lesser number of macrophages (Figure 2). The epidermis was hyperplastic with moderate orthokeratotic hyperkeratosis. No organisms typical for *Leishmania* sp. were found. In the kidney there was a moderate, multifocal interstitial inflammatory infiltrate consisting of lymphocytes and plasma cells surrounding the glomeruli and renal pelvis. There were also occasional small granulomas within the renal cortex. Within the myocardium, the gallbladder mucosa and periportally in the liver, there was multifocal infiltration of lymphocytes and plasma cells. There was lymphocytic vasculitis in few of the pancreatic venules. In the lymph nodes there was regressing follicular hyperplasia and massive numbers of plasma cells and macrophages extending from the medulla to the cortical area. Within some of the macrophages there were numerous 1–3 μm oval to round intracytoplasmic particles without obvious kinetoplasts (Figure 3). The particles were not clearly positive with the Giemsa stain. The bone marrow was hypercellular due to a marked number of plasma cells. In the spleen there was slight atrophy of the lymphoid tissue.

### Immunohistochemistry

**Figure 4:**
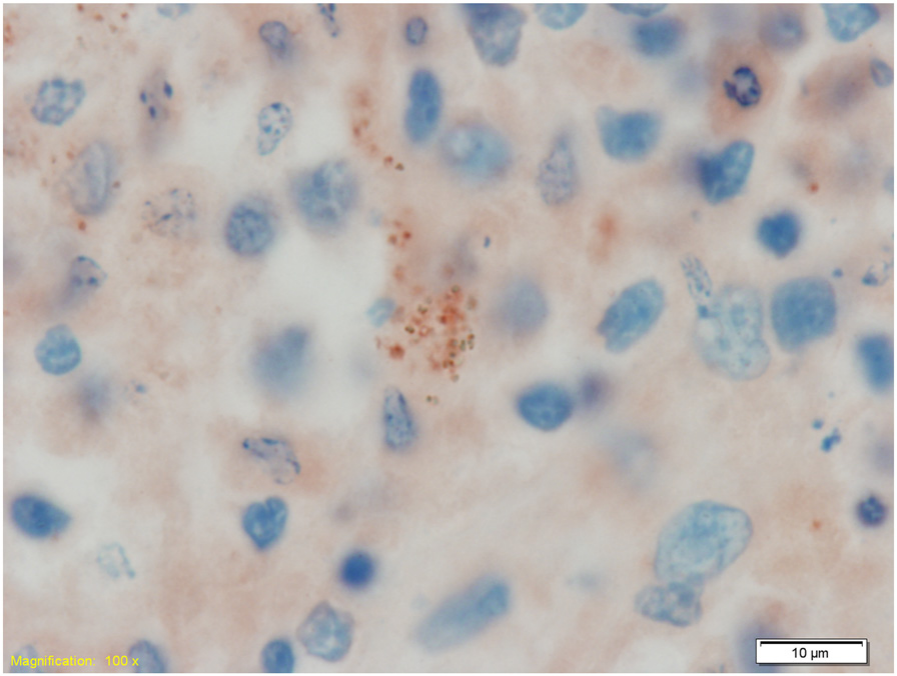
**Immunohistochemical staining of a lymph node from female D with antibodies against anti-kinetoplastid membrane protein-11 of** ***Leishmania*** **sp.** The misshapen particles within the macrophages were clearly positive.

Immunohistochemical staining for *Leishmania* sp. was performed on the skin of the ear pinnae, bone marrow, and selected lymph nodes of female D and the bone marrow of male E. Sections of 4 μm were stained using a UltraVision ONE HRP polymer detection system kit (Thermo Fisher Scientific, CA, USA). The sections were microwave heat-treated with pH 9 Dako Target retrieval Solution (Dako, Glostrup, Denmark) prior to incubation with antibodies. The sections were then incubated with primary monoclonal anti-kinetoplastid membrane protein-11 (Acris antibodies Inc., CA, USA) antibodies at a dilution of 1:100 for 120 min at room temperature. In the lymph nodes, the misshapen particles within the macrophages were strongly positive (Figure 4).

### PCR on tissues

PCR was performed on the skin of the pinnae of female D and the bone marrow of male E.

**Figure 5:**
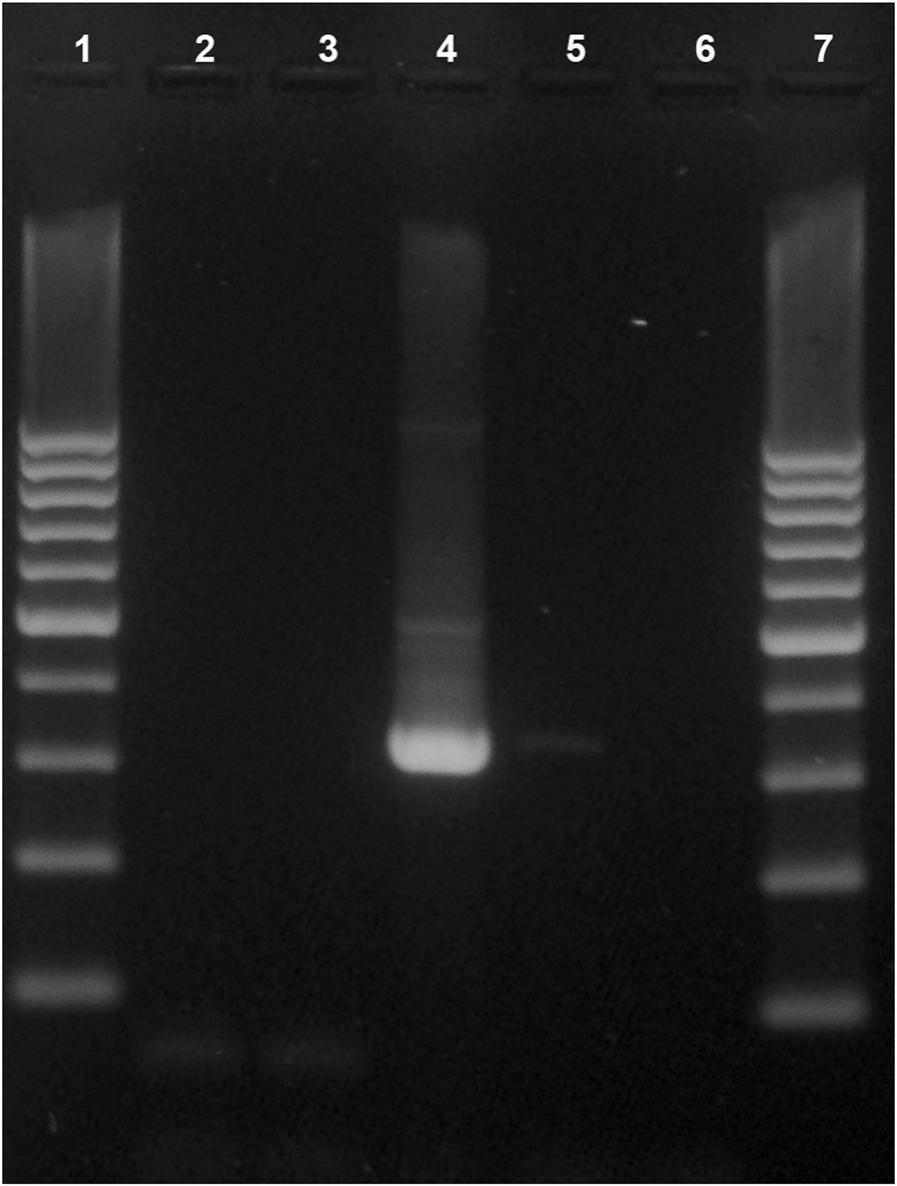
**Gel electrophoresis of the amplification products after** ***Leishmania*** **sp. 18S ribosomal RNA gene-nested PCR.** Lanes 1 and 7, 100 bp-marker; lanes 2–3, other diagnostic samples; lane 4, skin sample from female dog D with PCR product of 338 bp from Leishmania; lane 5, negative control sample; lane 6, negative reagent control.

DNA templates for nested-PCR were extracted from formalin-fixed or formalin-fixed paraffin-embedded tissue using a Blood and Tissue DNA extraction kit or an AllPrep DNA/RNA FFPE kit (Qiagen Inc, CA, USA). PCR reactions were carried out using a commercial kit (HotStarTaq Plus Master mix kit, Qiagen) according to the manufacturer’s instructions. The nested-PCR primers were designed to amplify 602 bp and 338 bp sized fragments of the *Leishmania* sp. 18S ribosomal RNA gene. The outer primer pair was slightly modified from da Silva [31]. The inner primer pair was designed based on the reference sequence from GeneBank (accession number GQ332359) (Table 1). The PCR products were visualized in an ethidium bromide agarose gel by UV light and a 100-bp marker (Gene Ruler™ 100 bp DNA ladder Plus, Fermentas) was used as a size reference. Skin and lymph nodes of female D (Figure 5) and bone marrow of male E were PCR positive. The identity of PCR products was confirmed by DNA sequencing (Eurofins MWG Operon, Germany).

**Table 1:** **Primer oligonucleotides used for nested PCR for** ***Leishmania*** **sp**

**Primer name**	**5′- 3′ primer sequence**	Position*	**Fragment**	**Reference**
LI1_F	GGTTCCTTTCCTGATTTACG	771–790	602 bp	da Silva [31]
LI1_R	GCCGGTAAAGGCCGAATAG	1372–1353		
LI2_F	GCAGTCATTTGACTTGAATTAG	853–875	338 bp	current paper
LI2_R	GGAAGGTATCCTTGAAGAATG	1190–1170		

*According to GeneBank accession no. GQ332359.

## Discussion and conclusions

*Leishmania* spp. are primarily transmitted via vectors in endemic areas, but may also spread transplacentally in pregnant females, via semen during mating and possibly also through bite wounds with blood to blood contact [14-16,18-20]. Compared to other dog breeds, boxer dogs are considered to be genetically more susceptible to leishmaniosis [13].

This is the first time that canine leishmaniosis has been confirmed in the Nordic countries as an autochthonous infection. This case shows that imported infected dogs pose a risk to domestic dogs regarding leishmaniosis. If leishmaniosis can spread via dog bites, as stated here, it may also be able to spread to humans if bitten by an infected dog.

Both dogs necropsied had manifested clinical symptoms typical for leishmaniosis, male E suffered from a chronic progressive kidney disease and female D showed classical signs of the cutaneous form [1,2,7,32]. Kidney disease is a common finding in leishmaniosis and in one study all *Leishmania*-positive dogs examined had histopathological changes in the glomeruli [33]. Chronic renal failure is considered to be the most common cause of death in leishmaniosis [34].

Chronic lymphoplasmacytic to histiocytic inflammation in the target tissues is a typical finding in this disease [1,7,32]. Usually, the *Leishmania* sp. organisms are numerous and clearly visible in the tissue sections by light microscopic examination; however, in more resistant individuals with an effective cell-mediated immune response, the number of organisms may be very low [1,7,32]. The number of organisms present is also related to the stage of the disease. Male E was not in the active stage of the infection and there were no organisms visible by light microscopy in the bone marrow; however, PCR, which is a more sensitive method, was positive for *Leishmania* sp. In the lymph nodes of female D there were a few macrophages that contained degenerate intracytoplasmic organisms that could not be identified as *Leishmania* sp. histologically. These misshapen organisms stained strongly positive with antibodies against leishmanial proteins. No organisms were seen in the skin samples of female D, but the skin was PCR positive for *Leishmania* sp. PCR appears to be a useful method in confirming the diagnosis in cases where there are no visible organisms.

The necropsy findings seem to indicate that the two dogs had mounted a Th1-type immune response, which is considered to be a *Leishmania*-resistant type of response, and which is associated with the alopecic form of the cutaneous disease and is consistent with a low number of organisms in the tissues [35]. The nodular or visceral forms are associated with poor immune response, large numbers of parasitic organisms in the tissues and severe progressive disease [1,12].

The origin of the infection appears to be male A that probably attracted the infection during his stay in Spain and subsequently developed severe clinical leishmaniosis. Dogs A, C and E received biting wounds during fighting and it seems likely that male E became infected at that incident. Female D could have contracted the disease venereally from the two males (A and E) or vertically from her mother (C). Since the vertical transmission rate is considered low [14,15], it seems more likely that the transmission was via infected semen. It has been speculated that *Leishmania* spp. might spread via bite wounds [20]. The history of events in our case suggests that this can indeed occur, and that transmission is possible even if the dogs involved show no clinical signs of the disease. All the previously reported cases of canine leishmaniosis in Finland have been in dogs imported from endemic areas [36-38].

It is likely that exotic diseases will be identified at increasing rates in Nordic countries in the future. Climate change may allow new insects to spread and survive in the Nordic countries and these insects may carry and spread new pathogens [39]. Travelling of dogs has become more and more commonplace, which increases their risk of contracting and spreading diseases [40]. The risk of spreading of the new vector-borne diseases within the Nordic countries has until now been considered low. Our findings show that this risk is not negligible and that leishmaniosis can spread in non-endemic areas without known vectors. In order to control this kind of risk, imported and breeding dogs should be tested for leishmaniosis before they leave their country of origin or before returning back home.
